# Colonoscopy overuse in colorectal cancer screening and associated factors in Argentina: a retrospective cohort study

**DOI:** 10.1186/s12876-017-0722-6

**Published:** 2017-12-15

**Authors:** Santiago Esteban, Ricardo Ricci, Sergio Terrasa, Karin Kopitowski

**Affiliations:** 1 Family and Community Medicine Division, Hospital Italiano de Buenos Aires, Tte. Gral. Perón, 4272 Ciudad Autónoma de Buenos Aires, Argentina; 2 Plan de Salud, Hospital Italiano de Buenos Aires, Ciudad Autónoma de Buenos Aires, Argentina; 3 Research Department, Instituto Universitario Hospital Italiano de Buenos Aires, Ciudad Autónoma de Buenos Aires, Argentina; 4 Research Department, Hospital Italiano de Buenos Aires, Ciudad Autónoma de Buenos Aires, Argentina; 5 Public Health Department, Instituto Universitario Hospital Italiano de Buenos Aires, Ciudad Autónoma de Buenos Aires, Argentina

**Keywords:** Colorectal cancer, Over screening, Colonoscopy

## Abstract

**Background:**

In recent years, there has been growing concern about the overuse of colonoscopy (CC). Our objective was to evaluate the incidence rate and cumulative probability of having a potentially inadequate CC (PI-CC, e.g. a CC that was performed earlier that recommended) and the association between the report of a hyperplastic polyp in the baseline CC report and the probability of having a PI-CC.

**Methods:**

A retrospective cohort of adults 50y/o or older with a complete baseline CC between January 1st and December 31st 2005, without reported lesions or with hyperplastic polyps, based on secondary data extracted from the electronic medical record of the Hospital Italiano of Buenos Aires. The outcome consisted of time until a PI-CC, defined as the time measured between basal colonoscopy and a colonoscopy performed earlier than the inter-screening interval recommended by the USPSTF and the USMSTF.

**Results:**

389 patients were included. The cumulative probability of receiving a PI-CC over 10 years was 0.29 (95% CI 0.241, 0.342). The incidence rate resulted in 30.91 PI-CC per 1000 person-years (95% CI 25.14, 38). The crude analysis of the association between the outcome and the presence of hyperplastic polyps in the baseline CC, showed a statistically significant difference between both groups (log rank, p 0.036). The multivariate analysis yielded a hazard ratio of 1.67 (95% CI 1.02–2.73).

**Conclusion:**

We observed that 3 in every 10 patients treated in our health system received a PI-CC during the first ten consecutive years after a normal complete CC. Furthermore, this could be in part attributed to the presence of a hyperplastic polyp in the baseline CC.

## Background

Colorectal cancer (CRC) screening has shown to decrease specific mortality from CRC [1–7] with adequate cost-effectiveness [8]. Screening guideless have been available since the early 2000s [9–12] with varying adoption rates [13–16]. Guidelines usually recommend a screening age range, a screening methodology and an adequate inter-screening interval that is dependent on the methodology used and the pathological findings. International guidelines overall agree on starting screening at age 50 for both women and men of average risk of developing colorectal cancer [17–20]. Stopping age is mostly recommended at 75 years (the European Guidelines [21] (EG) recommend stopping at 69); after that point and up to 85 years of age, the U.S. Preventive Services Task Force (USPSTF) [22] recommends individual decisions based on patient preference and life expectancy. Fecal occult blood testing (FOBT), rectosigmoidoscopy and colonoscopy (CC) are the most recommended methodologies for screening (except for the EG that recommend FOBT). There is also overall agreement that, if colonoscopy is the preferred method, inter-screening interval, when no lesions or hyperplastic polyps are found, is 10 years [17–20]. These recommendations have been standing with minor modifications for at least the last ten years. In Argentina, the USPSTF and the American Cancer Society (ACS) [17] guidelines are the most disseminated among general practitioners and gastroenterologists; also, local guidelines, that are in agreement with the ones mentioned before, have been available and updated since 2004 [23, 24].

In recent years, however, there has been growing concern about the overuse of colonoscopy, as different authors documented that screening intervals recommended in clinical practice guidelines are frequently not followed [25–28]. It is estimated that 16 to 27% of patients are overscreened [25, 26, 28] and that the median time to the next CC is 5.7 to 6.9 years when the recommended interval is ten [27].

Overscreening exposes patients to the unnecessary risks [29]of repeating a CC without a clear rationale, which in turn limits accessibility and increases waiting times for those who have a relevant indication. Furthermore, it dilapidates both economic and human resources, that once spent, are no longer available for other health interventions.

Previous studies [25, 27, 30] have identified predictors of CC overuse mostly related to the baseline CC such as the presence of hyperplastic polyps, the quality of colonic preparation, having received an incorrect screening recommendation by the professional who carried out the CC and having had the CC at a non-academic site. Knowing the presence, magnitude and potential causes or predictors of CC overuse for CCR screening is of vital importance for the design of interventions aimed at decreasing its magnitude. However, as of the date of this study, we did not find studies that had explored this problem in Argentina. Thus, we decided to evaluate the proportion of CC overuse in a cohort of adults who attended a University Hospital in Buenos Aires, Argentina and explore its predictors.

### Aims

To assess, in a cohort of adults with a complete baseline CC in 2005 without reported lesions or with hyperplastic polyps:

1. The incidence rate and cumulative probability of having a potentially inadequate CC (PI-CC).
2. The association between the report of a hyperplastic polyp in the baseline CC report and the probability of having a PI-CC.

## Methods

### Overview of study design and setting

The design is a retrospective cohort based on secondary data, extracted from the electronic medical record of the Hospital Italiano of Buenos Aires. The exposure was recorded prior to the occurrence of the result and this in turn was determined independently of the exposure.

The study was developed at the Hospital Italiano de Buenos Aires (HIBA), a high complexity university hospital. It has a private health insurance (Health Plan; HP-HIBA) that provides services to more than 150,000 members whose medical history can be tracked longitudinally through the hospital’s electronic medical records system (EMR).

The current rate of CRC screening in HP-HIBA affiliates 50 years old or older is approximately 56% [31]. All CCs at HIBA are performed by gastroenterologists or surgeons.

### Study population

Inclusion criteria: Adults, 50 years old or older, members of the HP-HIBA, who had a complete CC performed between January 1st and December 31st 2005 at the HIBA and without lesions or with hyperplastic polyps reported in the pathology report. A CC was considered complete if the endoscopist’s report described having been able to reach the cecum and that no significant amount of stools was present. If the patient had more than one CC performed that year, then the last one was considered as the baseline CC.

Exclusion criteria: history of CRC (inherited and non - inherited), familial adenomatous polyposis, inflammatory bowel disease or intestinal ischemia.

### Exposure

Patients were classified according to the presence of a hyperplastic polyp in the baseline CC (binary variable). This was performed by manually reviewing all CC and pathology reports.

### Outcome

The main outcome was defined as the time to a PI-CC, according to the criteria of the US Preventive Services Task Force [4] and the Multi-Society Task Force on Colorectal Cancer surveillance [17].

Appropriate CCs were considered those for which we were able to verify an acute reason in the EMR that justified the indication (bleeding, anemia, finding a primary lesion from metastases, abdominal pain, changes in bowel habits). PI-CC were those that were performed with an interval of less than 10 years from the baseline CC with an indication of screening (according to what was recorded by the requesting physician), or without the existence of an acute reason justifying it, in the six months prior to the date it was performed. To this end, all CC reports of the cohort members were manually assessed from 2006 onwards.

### Follow-up time

The follow-up started on the date of the baseline CC in 2005. Then, each patient was followed for a maximum of 10 years. Loss of follow-up, disenrollment from HP-HIBA, death or end of study was defined as censorship.

### Covariates

The variables gender, age at the time of baseline CC, reason for baseline CC (screening vs. non-screening), anemia, weight loss, and comorbidities were obtained from the EMR. The overall comorbidity burden was summarized by the Elixhauser comorbidity index, [32] excluding anemia and weight loss that were handled separately.

### Statistical analysis

The probability of receiving a PI-CE was estimated using the Kaplan-Meier method. The Cox model of proportional hazards was used for multivariate adjustment. The proportional hazards assumption was assessed by means of the scaled Schoenfeld residuals test and graph. Also, by analyzing the *log* − log(*S*_*t*_)*vs* log(*t*) graph for each variable. All analyses were performed using R (R Foundation for Statistical Computing, Vienna, Austria URL: http://www.cran.r-project.org/).

## Results

### Sample

**Fig. 1:**
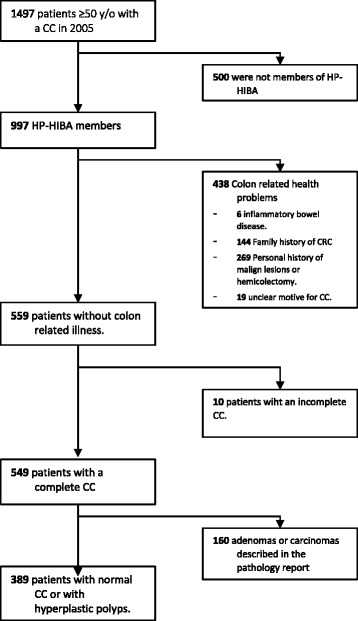
Patient flow chart

We identified 1497 patients who had a CC performed at HIBA during 2005. Of these, 997 were members of HP-HIBA. 438 patients were excluded because they met one or more of the exclusion criteria. Ten were excluded because their CC had been reported as incomplete. Of the remaining 549, 160 presented pathology reports describing lesions compatible with CRC, non-hyperplastic polyps, or colonic ulcers and were therefore also excluded. The patient flow chart is depicted in fig. 1.

A total of 161 colonoscopies were performed. For 71 of them, we were able to find a non-screening motive associated to the CC indication (digestive disorders (23; 32%), anemia (13; 18%), bleeding (14; 20%), others (4; 6%) and data registration errors (17; 24%)). Thus, we recorded them as appropriately indicated CCs. The remaining 90 CCs were PI because they were explicitly indicated for CRC screening or there was no acute motive in the EMR that justified them.

299 patients were censored (end of study: 199; loss of follow-up, disaffiliation or death: 100). Patient’s baseline characteristics are described in Table 1. The median follow-up time was 10 years (IQR: 4.75–10), the total follow-up person-years were 2911.62.

**Table 1:** Baseline characteristics of patients included in the cohort

	No Potentially inadequate colonoscopy	Potentially inadequate colonoscopy	*p*
N (total = 389)	299	90	
Age (median [IQR])	63.00 [58.00, 70.00]	62.00 [58.25, 65.00]	0.06
Age categories (%)			0.002
50–54	33 (11.0)	11 (12.2)	
55–59	71 (23.7)	19 (21.1)	
60–64	62 (20.7)	33 (36.7)	
65–69	56 (18.7)	19 (21.1)	
70–74	77 (25.8)	8 (8.9)	
Female (%)	200 (66.9)	53 (58.9)	0.20
No. comorbidities (%)			0.87
0	83 (27.8)	28 (31.1)	
1	100 (33.4)	27 (30.0)	
2	51 (17.1)	18 (20)	
3	40 (13.4)	11 (12.2)	
>3	25 (8.4)	6 (6.4)	
Baseline motive for Colonoscopy: Screening (%)	71 (23.7)	26 (28.9)	0.39
Reported polyp (%)	42 (14)	22 (24.4)	0.03
Polypectomy (%)	35 (11.7)	20 (22.2)	0.02
Mucosectomy (%)	1 (0.3)	3 (3.3)	0.06
Biopsy (%)	44 (14.4)	19 (21.1)	0.17
Anemia (%)	46 (15.4)	3 (3.3)	0.005
Weight loss (%)	3 (1.0)	0 (0.0)	0.79

### Cumulative probability and incidence density of a PI-CC

**Fig. 2:**
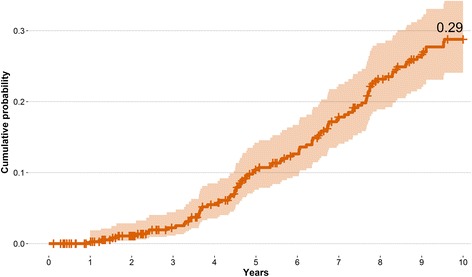
Cumulative probability of a potentially inadequate colonoscopy

Figure 2 shows the cumulative probability of receiving a PI-CC over 10 years, after a baseline CC with no lesions or hyperplastic polyps in patients without history of colonic illness (0.29, 95% CI 0.241, 0.342). The incidence rate resulted in 30.91 PI-CC per 1000 person-years (95% CI 25.14, 38).

### Association between the finding of a hyperplastic polyp in the baseline CC and a PI-CC

**Fig. 3:**
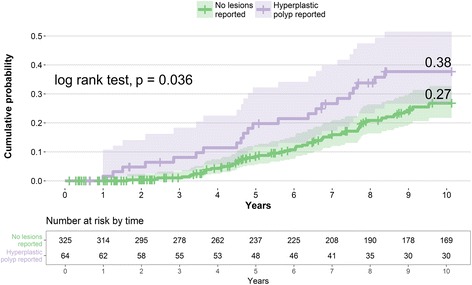
Cumulative probability of a PI-VCC by reported lesion in baseline VCC

Figure 3 plots the cumulative probability of having a PI-CC performed, according to whether a hyperplastic polyp had been reported in the 2005 CC. The crude analysis shows a statistically significant difference between both groups (log rank, p 0.036).

For the adjusted analysis, we used the model described in the methods section, which included the finding of a polyp in the baseline CC, sex, age at baseline CC, motive for CC indication, documentation of anemia before the baseline CC, the Elixhauser Comorbidity Index score and a previous history of weight loss. The variable age showed a nonlinear relationship with the result, thus it was included as a penalized cubic spline with two degrees of freedom.

We found no evidence of violation of the proportional hazards assumption.

The adjusted model showed that a patient with a baseline CC in which a hyperplastic polyp was reported has, on average, a 1.67-fold higher hazard (HR 1.67: 95% CI 1.02–2.73) of having a PI-CC performed compared to a patient in the no-polyp group, for the duration of follow-up. Table 2 describes the crude and adjusted hazard ratios.

**Table 2:** Crude and adjusted HR for having a PI-CC performed

Crude Hazard Ratio			
	HR	95% CI	*p*
Polyp reported	1.663	1.03, 2.69	0.04
Adjusted Hazard Ratio			
Polyp reported	1.67	1.02, 2.73	0.04
Sex: Female	0.81	0.53, 1.25	0.34
Age at baseline: linear term	0.979	0.945, 1.017	0.29
Age at baseline: non-linear terms	–	–	0.001
Motive for Colonoscopy: Screening	1.112	0.704, 1.758	0.65
Anemia	0.281	0.09, 0.895	0.03
Modified Elixhauser Comorbidity index	1.026	0.88, 1.2	0.74

## Discussion

Coinciding with previous studies published by Goodwin (7 years follow-up, 23.5%), [26] Schoen (7 years follow-up, 50.9%)[28] and Murphy(6 years follow-up, 16.4%),[25] we documented that 17.8% and 29% of the patients in our cohort had a PI-CC performed at 7 and 10 years after the baseline CC, respectively. We also found that the presence of hyperplastic polyps in the baseline CC report was associated with a higher probability of receiving a PI-CC. It is worth mentioning that Goodwin et al. [26] reported a cumulative probability of receiving a PI-CC of 23.5% after seven years for patients over 65 years old with no history of gastrointestinal cancer and a normal baseline CC. If we restrict our results to the same age-subgroup at seven years of follow-up, this value was much lower (10.7% at 7 years, 16.2% at 10 years). However, when assessing the time to PI-CC by type of finding in baseline CC, Kruse et al. [7] described shorter intervals than those in our study (Kruse et al. vs this study; CC without polyps: 6.9 years [IQR 5.1–10] years vs 10 [IQR 4.7–10]; CC with hyperplastic polyps: 5.7 years [IQR 4.9–9.7] vs. 8.38 years [IQR 5.04–10]). In addition, and unlike us, these authors found no association between the report of a hyperplastic polyp in the baseline CC and an incident PI-CC (OR 0.61 95% CI 0.3–1.22). On the other hand, Johnson et al. [10] report an association between hyperplastic polyps and a PI-CC consistent with our findings (OR 3.1 95% CI 1.7–5.5). In our case, it was impossible to include the endoscopist’s recommendation in the model, since the CC reports do not include this data in our hospital.

These data shed light on a problem of great complexity such as the phenomenon of overuse. Also, we propose a possible causal determinant (or at least a predictor) for the anticipated indication of a surveillance CC, as is the presence of hyperplastic polyps. However, many of the causal factors that determine the anticipated CC probably exceed the model proposed in our study. Many of the patients’ determining factors are probably not adequately captured in the EMR (eg, anxiety, fear of cancer, “heavy” user profile, etc.). Neither are factors related to the treating physician or the health system. It is worth noting that, in a previous study conducted at our institution, nearly 20% of health professionals thought that patients with hyperplastic polyps should have a new CC performed earlier than ten years, [33] which is consistent with the findings we are reporting. Despite these limitations, our study presents the first data on this problem in South America.

Like most preventive health interventions, CRC screening strategies have benefits and harms. That is, why when implementing a CRC screening program, one must ensure that there is total net benefit. And for this, it is essential to maximize benefits and minimize harms. In order to maximize the benefits, we must ensure that the CC is performed by properly trained personnel, that the patients have adequate colonic preparation, that the equipment is in good condition, that the biopsies are analyzed by excellent pathologists, etc. Strategies to minimize harm in screening programs involve combining carefully chosen start and discontinuation variables and an optimal inter-screening interval. The alteration of these parameters can dangerously tip this delicate balance in favor of net harm. In the case of CRC screening, a shorter-than-recommended CC interval, can account for most of the damage described for screening in general (physical and psychological harms, unnecessary expenses and opportunity costs) [34].

On the other hand, it is important to keep in mind that, although CRC screening has shown to decrease CRC specific mortality and some cohort studies show an effect on incidence-based mortality, [35] so far, no meta-analyses of RCTs or individual RCTs of any CRC screening modalities have shown a reduction in all-cause mortality [22, 36–38]. This could be due to the fact that newer screening methods, like fecal immunochemical testing or colonoscopy, have not been evaluated so far in terms of their effect on mortality, although several RCTs are ongoing. [39–43]. Also, it is important to remember that assessing causal effects on overall-mortality is a rather difficult task due to co-interventions affecting the outcome in long follow-up longitudinal studies, such as are needed to assess this type of outcome. Still, the best evidence available at the moment does not indicate that CRC screening reduces overall-mortality. This is particularly important since reductions in mortality from the disease being screened (in this case CRC) may be offset by an increase in deaths due to the effects of the diagnostic cascade originated from the same screening programs. Such “out-of-target deaths” are particularly likely when screening leads to diagnostic cascades with potential damage. If an inadequate interval is added to this, the potential damage is even greater. This would run the risk of falling into the paradox that the screening of a disease would reduce mortality from that cause and increase overall mortality at the expense of deaths caused by the same screening program. Furthermore, the potential damages that stem from longer waiting times for patients who adequately need a CC and the dilapidation of health resources which will no longer be spent on other strategies with demonstrated cost-effectiveness, help add more complexity to an already very complex problem.

## Conclusion

We documented in this investigation that one every three patients treated in our health system receive a PI-CC during the first ten consecutive years after a normal complete CC. Furthermore, this could be in part attributed to the presence of a hyperplastic polyp in the baseline CC. This information will allow us to improve on our current CRC screening programs in the search for minimizing the potential damages and maximizing its net benefits.

## Abbreviations

CC: Colonoscopy
CRC: Colorectal cancer
EHR: Electronic Health Records
EMR: Electronic Medical Records
HIBA: Hospital Italiano de Buenos Aires
HP-HIBA: Health Plan at Hospital Italiano de Buenos Aires
PI-CC: Potentially-inadequate colonoscopy
